# Atopic diseases/diathesis and subsequent ischemic stroke among patients with bipolar disorder: A nationwide longitudinal study

**DOI:** 10.1371/journal.pone.0200682

**Published:** 2018-08-16

**Authors:** Li-Chi Chen, Mu-Hong Chen, Tung-Ping Su, Shih-Jen Tsai, Ya-Mei Bai, Cheng-Ta Li, Albert C. Yang, Wen-Han Chang, Tzeng-Ji Chen

**Affiliations:** 1 Department of Psychiatry, Taipei Veterans General Hospital, Taipei, Taiwan; 2 Department of Psychiatry, Cheng Hsin General Hospital, Taipei, Taiwan; 3 Division of Psychiatry, School of Medicine, National Yang-Ming University, Taipei, Taiwan; 4 Department of Family Medicine, Taipei Veterans General Hospital, Taipei, Taiwan; 5 Institute of Hospital and Health Care Administration, National Yang-Ming University, Taipei, Taiwan; Federico II University of Naples, ITALY

## Abstract

**Introduction:**

Previous studies have suggested that both bipolar disorder and atopy are associated with an increased risk of stroke. However, the role of atopic diseases/diathesis in the risk of stroke among patients with bipolar disorder remains unclear.

**Methods:**

Using Taiwan’s National Health Insurance Research Database, we selected 55,593 patients with bipolar disorder between 2002 and 2008, divided them into patients with atopic diseases/diathesis (n = 21,050) and patients without atopic diseases/diathesis (n = 34,543), and observed them until the end of 2011. Patients who experienced a stroke during the follow-up period were identified.

**Results:**

Patients with bipolar disorder and atopic diseases/diathesis had an elevated risk of ischemic stroke (hazard ratio [HR]: 1.44, 95% confidence interval [CI]: 1.25–1.59) compared with patients with only bipolar disorder; a dose-dependent relationship was observed between the number of allergic comorbidities and the risk of ischemic stroke (1 atopic disease, HR: 1.30, 95% CI: 1.13–1.49; 2 atopic diseases, HR: 1.59, 95% CI: 1.33–1.91; ≥ 3 atopic diseases, HR: 2.09, 95% CI: 1.50–2.91).The role of atopic diseases in the risk of hemorrhagic stroke among patients with bipolar disorder was nonsignificant (HR: 0.84, 95% CI: 0.64–1.09).

**Conclusions:**

Patients with bipolar disorder and atopic diseases/diathesis are more prone to ischemic stroke later in life than are those without atopic diseases/diathesis. Further study is required to investigate the underlying mechanism linking atopy, bipolar disorder, and stroke.

## Introduction

The high prevalence of stroke-related risk factors, including hypertension, dyslipidemia, and diabetes mellitus, among patients with bipolar disorder has gained much attention in the public health and psychiatric fields with respect to its subsequent cerebrovascular diseases [1]. Mounting evidence has suggested that bipolar disorder is associated with death from coronary heart disease and stroke [2, 3]. Lin et al. reported that the risk of cerebrovascular accidents was 2.1-fold to 3.3-fold higher among patients with bipolar disorder than among controls [4]. One systematic metaanalysis determined that patients with bipolar disorder had an elevated likelihood of stroke (relative risk [RR]: 1.74, 95% confidence interval [CI]: 1.29–2.35) compared with subjects in a control group [5].

Because bipolar disorder is a multisystemic disorder affecting cardiovascular status and immunological function [3, 6, 7], previous studies have investigated the association between atopic diseases/diathesis and bipolar disorder [8–10]. For example, Goodwin et al. reported that patients with bipolar disorder had an increased risk of atopic disease (odds ratio [OR]: 2.0, 95% CI: 1.0–3.8) and lifetime asthma (OR: 5.64, 95% CI: 1.95–16.35) [8, 9]. Furthermore, studies have suggested that chronic systemic inflammation may lead to cerebrovascular thrombotic events and stroke [11–13]. A growing body of evidence suggests that atopic diseases, including asthma, allergic rhinitis, and atopic dermatitis, are chronic systemic inflammatory diseases that may further increase the likelihood of stroke-related risk factors and stroke [14–16]. Because the comorbidity of bipolar disorder and atopy is common and both conditions are associated with an increased risk of stroke, the role of atopic diseases/diathesis in the association between bipolar disorder and stroke warrants investigation.

Using a longitudinal study design and a large sample extracted from Taiwan’s National Health Insurance Research Database (NHIRD), we investigated the impact of atopic comorbidity on the risk of stroke among patients with bipolar disorder. We hypothesized that patients with bipolar disorder and atopic comorbidity have an increased risk of stroke later in life compared with patients with only bipolar disorder.

## Methods

### Data source

The National Health Insurance program was inaugurated in 1995 and covers approximately 99% of Taiwan’s 23 million residents (http://www.nhi.gov.tw/). The NHIRD is administered by the National Health Research Institute and provides comprehensive patient information such as demographic data, clinical visit dates, and disease diagnoses. The identities of all patients included in the NHIRD are withheld to ensure privacy. The diagnostic codes used were based on the International Classification of Diseases, Ninth Revision, Clinical Modification (ICD-9-CM). The NHIRD has been used extensively in many epidemiologic studies in Taiwan [17–20]. Written consent from study patients was not obtained because the NHI dataset consists of de-identified secondary data for research purposes, and the Institutional Review Board of Taipei Veterans General Hospital issued a formal written waiver for the need for consent. This study was approved by the ethical review board of the Taipei Veterans General Hospital (approval number: 2014-10-009CC).

### Inclusion criteria for patients with bipolar disorder and with or without atopic diseases

Patients who were diagnosed with bipolar disorder (ICD-9-CM code: 296.0, 296.1, 296.4–296.7, 296.80, 296.81, 296.89) by psychiatrists between January 1, 2002 and December 31, 2008, and who had no history of stroke (ICD-9-CM codes: 430–438) were included in our study. We divided these patients into 2 subgroups according to the presence or absence of atopic diseases/diathesis. Atopic diathesis was defined as diagnosed asthma, allergic rhinitis, atopic dermatitis, and allergic conjunctivitis. Asthma (ICD-9-CM code: 493) was diagnosed by emergency room doctors, internists, pulmonologists, or rheumatologists; allergic rhinitis (ICD-9-CM code: 477) was diagnosed by family medicine physicians, internists, pulmonologists, rheumatologists, or otolaryngologists; atopic dermatitis (ICD-9-CM codes: 691 or 691.8) was diagnosed by dermatologists; and allergic conjunctivitis (ICD-9-CM: 372.05, 372.10, and 372.14) was diagnosed by ophthalmologists. We observed patients until death or December 31, 2011, identifying patients who had an ischemic stroke (ICD-9-CM codes: 433–435) or hemorrhagic stroke (ICD-9-CM: 430–432) that was diagnosed by neurologists, neurosurgeons, or emergency room doctors after a neuroimaging examination. In addition, we assessed the association between other stroke-related risk factors, including hypertension, dyslipidemia, diabetes mellitus, head injury, and chronic renal diseases, with the risk of ischemic stroke and hemorrhagic stroke. All diagnoses were made at least twice by corresponding physicians to ensure diagnostic validity. Moreover, level of urbanization (Level 1 [most urbanized area] to Level 5 [least urbanized area]) was assessed in our study [21].

### Statistical analysis

For between-group comparisons, the independent *t* test was used for continuous variables and Pearson’s X^2^ test for nominal variables when appropriate. Two Cox regression models were used to investigate the hazard ratios (HRs) and 95% CIs of ischemic stroke and hemorrhagic stroke. The primary model was applied to investigate the presence or absence of atopic diseases/diathesis as a categorical variable with the risks of ischemic stroke and hemorrhagic stroke; the secondary model used the number of atopic comorbidities as a categorical variable to assess the risks of ischemic stroke and hemorrhagic stroke. The 2 models were adjusted according to demographic factors and medical comorbidities. A 2-tailed p value of less than 0.05 was considered statistically significant. All data processing and statistical analyses were performed using Statistical Package for Social Science (SPSS) Version 17 (SPSS Inc.) and Statistical Analysis Software (SAS) Version 9.1 (SAS Institute, Cary, NC).

## Results

In total, 55,593 patients with bipolar disorder between 2002 and 2008 were identified and divided into 2 subgroups according to the presence (n = 21,050) or absence (n = 34,543) of atopic diseases/diathesis (Table 1). Most patients with bipolar disorder and comorbid atopic diseases/diathesis were female (63% vs. 53.2%, p < 0.001), and more resided in urbanized areas (p < 0.001) and had a lower income (p = 0.003) than did patients with bipolar disorder alone (Table 1). During the follow-up period, patients with bipolar disorder and atopic diseases/diathesis had an elevated incidence of ischemic stroke (2.5% vs. 1.6%, p < 0.001) and an equal incidence of hemorrhagic stroke (0.4% vs. 0.5%, p = 0.456) compared with patients without atopic diseases/diathesis (Table 1). Furthermore, the prevalence of medical comorbidities, including hypertension (19.8% vs. 15.5%, p < 0.001), dyslipidemia (17.1% vs. 11.3%, p < 0.001), diabetes mellitus (12.0% vs. 9.6%, p < 0.001), and chronic renal disease (2.5% vs. 1.8%, p < 0.001), was significantly higher in the atopic subgroup than in the nonatopic subgroup (Table 1).

**Table 1:** Demographic data and incidence of stroke of patients with bipolar disorder.

	Bipolar disorder (n = 55593)		
	with atopic diathesis (n = 21050)	without atopic diathesis (n = 34543)	*p*-value
Age at diagnosis/enrollment (years, SD; n, %)	37.69 (15.60)	38.12 (14.49)	0.001
<40	12580 (59.8)	20202 (58.5)	
40~59	6423 (30.5)	11524 (33.4)	
≧60	2047 (9.7)	2817 (8.2)	
Sex (n, %)			<0.001
Male	7797 (37.0)	16179 (46.8)	
Female	13253 (63.0)	18364 (53.2)	
Ischemic stroke (n, %)	522 (2.5)	542 (1.6)	<0.001
Age at ischemic stroke (years, SD)	59.74 (14.14)	59.04 (13.98)	0.421
Hemorrhagic stroke (n, %)	86 (0.4)	157 (0.5)	0.456
Age at hemorrhagic stroke (years, SD)	53.13 (15.72)	51.71 (16.17)	0.511
Atopic comorbidities (n, %)			
0	-	34543 (100)	-
1	14678 (69.7)	-	
2	5277 (25.1)	-	
≧3	1095 (5.2)	-	
Average (n, SD)	1.36 (0.59)	-	-
Medical comorbidities (n, %)			
Hypertension	4170 (19.8)	5356 (15.5)	<0.001
Dyslipidemia	3596 (17.1)	3915 (11.3)	<0.001
Diabetes mellitus	2522 (12.0)	3320 (9.6)	<0.001
Chronic renal diseases	528 (2.5)	605 (1.8)	<0.001
Level of urbanization (n, %)			<0.001
1 (most urbanized)	5536 (26.3)	8175 (23.7)	
2	7034 (33.4)	11204 (32.4)	
3	2263 (10.8)	3959 (11.5)	
4	2384 (11.3)	4463 (12.9)	
5 (most rural)	3833 (18.2)	6742 (19.5)	
Income-related insured amount (n, %)			0.003
≤ 15,840 NTD/month	10444 (49.6)	17156 (49.7)	
15,841~25,000NTD/month	6699 (31.8)	11336 (32.8)	
≥ 25,001NTD/month	3907 (18.6)	6051 (17.5)	

SD: standard deviation; NTD: new Taiwan dollar.

The Cox regression analysis with adjustments for demographic factors and medical comorbidities revealed that, compared with patients with bipolar disorder and without atopic diseases/diathesis, patients with bipolar disorder and atopic diseases/diathesis had an increased likelihood of ischemic stroke (HR: 1.41, 95% CI: 1.25–1.59); a dose-dependent relationship was observed between the number of allergic comorbidities and the risk of ischemic stroke (1 atopic disease, HR: 1.30, 95% CI: 1.13–1.49; 2 atopic diseases, HR: 1.59, 95% CI: 1.33–1.91; ≥ 3 atopic diseases, HR: 2.09, 95% CI: 1.50–2.91) (Table 2). In addition, owing to the lack of current evidence between allergic conjunctivitis and stroke, the additional analysis removing allergic conjunctivitis in the atopic sample found a consistent finding that a dose-dependent relationship was observed between the number of allergic comorbidities and the risk of ischemic stroke (1 atopic disease, HR: 1.27, 95% CI: 1.09–1.48; ≥2 atopic diseases, HR: 1.57, 95% CI: 1.25–1.98).

**Table 2:** Cox regression models for ischemic stroke among patients with bipolar disorder (adjusted for demographic data and medical comorbidities).

	Stratified by age			Stratified by sex		
	<40 years (HR, 95%CI)	40~59 years (HR, 95%CI)	≧60 years (HR, 95%CI)	Male (HR, 95%CI)	Female (HR, 95%CI)	Total (HR, 95%CI)
	Ischemic stroke					
Atopic comorbidities, presence vs. absence	**1.73 (1.23~1.41)**	**1.56 (1.30~1.87)**	**1.28 (1.07~1.55)**	**1.34 (1.12~1.60)**	**1.49 (1.26~1.76)**	**1.41 (1.25~1.59)**
1	**1.48 (1.01~2.18)**	**1.35 (1.09~1.67)**	**1.27 (1.04~1.57)**	**1.23 (1.01~1.50)**	**1.38 (1.14~1.67)**	**1.30 (1.13~1.49)**
2	**2.34 (1.45~3.77)**	**1.90 (1.44~2.50)**	1.28 (0.97~1.70)	**1.56 (1.19~2.05)**	**1.63 (1.28~2.09)**	**1.59 (1.33~1.91)**
≧3	2.32 (0.93~5.80)	**2.80 (1.80~4.35)**	1.32 (0.72~2.42)	**1.95 (1.14~3.35)**	**2.24 (1.47~3.42)**	**2.09 (1.50~2.91)**
Medical comorbidities, presence vs. absence						
Hypertension	**2.68 (1.72~4.17)**	**1.48 (1.21~1.81)**	**1.21 (0.99~1.48)**	**1.63 (1.33~2.00)**	**1.26 (1.03~1.54)**	**1.44 (1.25~1.66)**
Dyslipidemia	0.92 (0.55~1.56)	0.86 (0.68~1.07)	0.75 (0.60~1.14)	1.05 (0.84~1.30)	0.80 (0.65~1.08)	0.91 (0.78~1.06)
Diabetes mellitus	**1.64 (0.94~2.86)**	**1.37 (1.09~1.71)**	**1.25 (1.02~1.54)**	**1.23 (1.01~1.54)**	**1.62 (1.33~1.98)**	**1.43 (1.23~1.65)**
Chronic renal diseases	0.81 (0.20~3.32)	0.87 (0.54~1.43)	1.30 (0.95~1.78)	0.96 (0.66~1.39)	1.21 (0.84~1.73)	1.08 (0.84~1.40)

HR: hazard ratio; CI: confidence interval. **Bold** type indicates the statistical significance.

Stratified by sex and age group, the Cox regression models also revealed that both male (HR: 1.34, 95% CI: 1.12–1.60) and female (HR: 1.49, 95% CI: 1.26–1.76) patients with bipolar disorder and atopic diseases/diathesis were prone to ischemic stroke during the follow-up period, and 3 age groups (< 40 years, HR: 1.73, 95% CI: 1.23–1.41; 40–59 years, HR: 1.56, 95% CI: 1.30–1.87; and ≥ 60 years, HR: 1.28, 95% CI: 1.06–1.54) of patients with bipolar disorder and atopic diseases/diathesis had an increased risk of ischemic stroke (Table 2).

Allergic diseases had no significant role in the risk of hemorrhagic stroke (HR: 0.84, 95% CI: 0.64–1.09) among patients with bipolar disorder, whereas hypertension (HR: 1.48, 95% CI: 1.09–2.02) was associated with an elevated risk of hemorrhagic stroke (Table 3).

**Table 3:** Cox regression models for hemorrhagic stroke among patients with bipolar disorder (adjusted for demographic data and medical comorbidities).

	Stratified by age			Stratified by sex		
	<40 years (HR, 95%CI)	40~59 years (HR, 95%CI)	≧60 years (HR, 95%CI)	Male (HR, 95%CI)	Female (HR, 95%CI)	Total (HR, 95%CI)
	Hemorrhagic stroke					
Atopic comorbidities, presence vs. absence	0.79 (0.48~1.30)	1.13 (0.76~1.69)	0.65 (0.39~1.09)	0.74 (0.52~1.07)	1.01 (0.68~1.50)	0.84 (0.64~1.09)
1	0.83 (0.48~1.45)	1.23 (0.84~1.98)	0.74 (0.42~1.29)	0.91 (0.62~1.33)	1.03 (0.66~1.60)	0.94 (0.70~1.25)
2	0.83 (0.32~1.94)	0.86 (0.41~1.80)	0.33 (0.10~1.05)	0.35 (0.14~1.86)	1.02 (0.54~1.90)	0.63 (0.38~1.04)
≧3	-	0.49 (0.07~3.52)	1.31 (0.33~5.71)	0.39 (0.06~2.84)	0.79 (0.19~3.23)	0.57 (0.18~1.78)
Medical comorbidities, presence vs. absence						
Hypertension	1.18 (0.56~2.50)	**2.04 (1.34~3.10)**	0.96 (0.57~1.60)	**1.64 (1.10~2.43)**	1.26 (0.77~2.04)	**1.48 (1.09~2.02)**
Dyslipidemia	0.45 (0.15~1.33)	0.73 (0.44~1.20)	0.51 (0.26~1.01)	0.68 (0.42~1.11)	0.58 (0.33~1.03)	0.65 (0.45~1.04)
Diabetes mellitus	1.18 (0.44~3.16)	1.03 (0.62~1.69)	0.98 (0.55~1.75)	1.23 (0.78~1.94)	0.88 (0.50~1.55)	1.08 (0.76~1.54)
Chronic renal diseases	2.51 (0.60~10.43)	0.57 (0.14~2.31)	**2.60 (1.31~5.19)**	1.39 (0.64~3.02)	2.11 (0.96~4.66)	1.63 (0.94~2.84)

HR: hazard ratio; CI: confidence interval. **Bold** type indicates the statistical significance.

## Discussion

This is the first study to investigate the impact of atopic comorbidities to the risk of stroke among patients with bipolar disorder. The findings support our hypothesis that after adjustment for demographic factors and stroke-related medical comorbidities, patients with bipolar disorder and atopic diseases/diathesis have an increased risk of ischemic stroke later in life; a dose-dependent relationship exists between the number of atopic comorbidities and the likelihood of ischemic stroke.

Previous studies have reported a frequent comorbid association between bipolar disorder and atopic diseases. Jerrell et al. revealed that more than one-fifth of patients with bipolar disorder had comorbid atopic diseases and atopic diathesis [22]. In a sample of 4,181 adults, Goodwin et al. reported that atopy was associated with an increased prevalence of bipolar disorder (OR: 2.0, 95% CI: 1.0–3.8) [8]. Furthermore, a growing body of evidence has suggested that both bipolar disorder and atopic diseases are related to stroke-related comorbidities and subsequent ischemic stroke [23–28]. Schanen et al. noted that after adjustment for blood pressure, lipid profile, and diabetes diagnosis, asthma was an independent risk factor for incident stroke (HR: 1.50, 95% CI: 1.04–2.15) [16]. A recent metaanalysis of 27,092 patients with bipolar disorder determined that the risk of stroke among patients with bipolar disorder was significantly high (RR: 1.74, 95% CI: 1.29–2.35) [5]. Chen et al. reported that patients with bipolar disorder and atopic comorbidity were prone to stroke-related diseases, including hypertension, dyslipidemia, and diabetes mellitus [29]. In accordance with previous studies, our study determined that patients with bipolar disorder and atopic diseases/diathesis had a higher prevalence of metabolic disorders and an increased risk of ischemic stroke than did those with only bipolar disorder. A dose-dependent increase in the risk of ischemic stroke among patients with bipolar disorder and atopic comorbidities was still present after adjustment for demographic factors and medical comorbidities.

An atopy-related immunological disturbance may explain the association between bipolar disorder and ischemic stroke. Previous studies have suggested that the proinflammatory cytokines related to recurrent atopic responses, including interleukin (IL)-1, IL-6, and tumor necrosis factor (TNF)-α, play a critical role in the pathophysiology of stroke-related risk factors (atherosclerosis and hypertension) and ischemic stroke [30–34]. In addition, some systematic review studies have suggested the possibility of an alteration in several inflammatory cytokines, including TNF-α and IL-6, among patients with bipolar disorder [35–37]. Furthermore, the effect of glycogen synthase kinase-3 (GSK-3) dysregulation has been proposed as an explanation for the associations among the pathophysiologies of bipolar disorder, atopy, and ischemic stroke [38–40]. GSK-3, a proapoptotic enzyme in many critical intracellular signaling mechanisms, has been demonstrated to modulate the inflammatory process and cell apoptosis [41, 42]. Previous studies have proposed that abnormal expression, activity, and signaling systems connected to GSK-3β contributed to the pathophysiology of bipolar disorder [40, 42, 43], and may influence the atopic inflammatory response [40, 44, 45]. Our study revealed that atopic comorbidities have a significant role in the risk of ischemic stroke among patients with bipolar disorder. However, further study is required to clarify the underlying mechanisms of overlapping proinflammatory cytokines and GSK-3β dysregulation in bipolar disorder and the atopic comorbidities in susceptibility to ischemic stroke.

In accordance with previous findings that bipolar disorder and atopic diseases were unassociated with hemorrhagic stroke, our study discovered no evidence of an association between atopic diseases/diathesis and hemorrhagic stroke among patients with bipolar disorder [5, 46]. However, as mentioned, both bipolar disorder and atopic diseases increased the likelihood of hypertension, which is a significant risk factor for hemorrhagic stroke [1, 47–49]. A possible cascade from bipolar disorder and allergic diseases to hypertension and hemorrhagic stroke requires further investigation.

This study had several limitations. First, the incidence of stroke and the prevalence of bipolar disorder and atopic diseases may have been underestimated because only patients who sought medical assistance were examined in our study. However, all patients in our study received diagnoses from board-certified physicians, yielding a high diagnostic validity. Second, we did not investigate the effects of medications for bipolar disorder and atopic diseases on the risk of stroke because of the prohibitive complexity posed by medication use during such a long follow-up period. Third, we were unable to examine the influence of factors not included in the NHIRD patient information, such as age at onset of bipolar disorder, the subtypes of bipolar disorder, family history, personal lifestyle, obesity, and environmental factors.

In conclusion, after adjusting for demographic factors and stroke-related medical comorbidities, we determined that patients with bipolar disorder and atopic diseases/diathesis have an increased likelihood of ischemic stroke later in life compared with patients with only bipolar disorder. In addition, we observed a dose-dependent relationship between the number of atopic comorbidities and the risk of ischemic stroke among patients with bipolar disorder. In the clinical practice, we suggest that clinicians should pay more attention to the likelihood of metabolic and cerebrocardiovascular diseases among patients with bipolar disorder, especially those with atopic diseases. A high-quality and integrated care with detection and follow-up of cerebrocardiovascular risk factors such as smoking, hypertension, obesity or unhealthy life styles should be necessary in patients with bipolar disorder and atopic diseases. Further study is required to investigate the underlying mechanism linking bipolar disorder, atopic diseases/diathesis, and ischemic stroke, and to elucidate whether prompt intervention in atopic diseases may reduce the risk of ischemic stroke among patients with bipolar disorder.

## Supporting information

S1 Data Data of the atopy group and the non-atopy group. (XLSX)
